# Synergistic activity of the combination of falcarindiol and itraconazole *in vitro* against dermatophytes

**DOI:** 10.3389/fcimb.2023.1128000

**Published:** 2023-05-03

**Authors:** Siyue Kan, Jingwen Tan, Qing Cai, Lulu An, Zhiqin Gao, Hong Yang, Siyu Liu, Risong Na, Lianjuan Yang

**Affiliations:** ^1^ Department of Medical Mycology, Shanghai Skin Disease Hospital, Tongji University School of Medicine, Shanghai, China; ^2^ College of Plant Protection, Henan Agricultural University, Zhengzhou, China

**Keywords:** falcarindiol, itraconazole, dermatophytes, synergistic, additive, polyacetylene alcohols

## Abstract

Previous studies have shown that natural polyacetylene alcohols, such as falcarindiol (FADOH), have good antifungal effects on plant fungi. While its effect on fungi that infect humans remains to be explored. In our study, checkerboard microdilution, drop-plate assay, and time-growth method were employed to analyze the interactions between FADOH and itraconazole (ITC) *in vitro* against dermatophytes, including 12 *Trichophyton rubrum* (*T. rubrum*), 12 *Trichophyton mentagrophytes* (*T. mentagrophytes*), and 6 *Microsporum canis* (*M. canis*). The results showed that the combination of FADOH and ITC exhibited synergistic and additive activity against 86.7% of all tested dermatophytes. FADOH had an excellent synergistic effect on ITC against *T. rubrum* and *T. mentagrophytes*; the synergistic rates were 66.7% and 58.3%, respectively. On the contrary, FADOH combined with ITC showed poor synergistic inhibitory activity (16.7%) against *M. canis*. Moreover, the additive rates of these two drugs against *T. rubrum*, *T. mentagrophytes*, and *M. canis* were 25%, 41.7%, and 33.3%, respectively. No antagonistic interactions were observed. The drop-plate assay and time-growth curves confirmed that the combination of FADOH and ITC had a potent synergistic antifungal effect. The *in vitro* synergistic effect of FADOH and ITC against dermatophytes is reported here for the first time. Our findings suggest the potential use of FADOH as an effective antifungal drug in the combined therapy of dermatophytoses caused especially by *T. rubrum* and *T. mentagrophytes*.

## Introduction

Dermatophyte infections are often superficial and involve the epidermis, skin and keratinized structures like hairs and nails (Akay et al., 2019). On rare occasions, dermatophytes can cause invasive infections involving hair follicles, subcutaneous tissues, and internal organs such as lymph nodes, bones, and the brain (Wang et al., 2021; Silva et al., 2022). Invasive dermatophytoses can be classified as Majocchi’s granuloma, deep dermatophytosis, pseudomycetoma, and disseminated dermatophytosis (Rouzaud et al., 2015; Wang et al., 2021; Silva et al., 2022). A systematic review of invasive dermatophyte infections showed that *Trichophyton rubrum* (*T. rubrum*) was the most prevalent pathogen responsible for invasive dermatophytoses, followed by *Trichophyton mentagrophytes* (*T. mentagrophytes*), and *Microsporum canis* (*M. canis*) (Wang et al., 2021). Deep dermatophytosis is a rare and sometimes life-threatening fungal infection. This condition mainly occurs in immunosuppressed individuals or patients with mutations in genes, including Caspase Recruitment Domain-containing protein 9 (CARD9) and signal transducer and activator of transcription 3 (STAT3) (Wang et al., 2021). Therefore, treating chronic superficial tinea in these immunosuppressed patients and patients with genetic mutations is critical to prevent the development of invasive dermatophytoses.

Although dermatophytoses are generally considered easy to treat, dermatophyte resistance to existing antifungal drugs (e.g., terbinafine, itraconazole, fluconazole, amphotericin B, and posaconazole) is increasing, and infections are more likely to recur(Khurana et al., 2019; Wang et al., 2021). More importantly, these drugs are usually considered to cause some side effects, such as liver function damage. To overcome the limitations of current treatment strategies, it is necessary to explore combination therapies through *in vitro* experiments.

**Figure 1:**
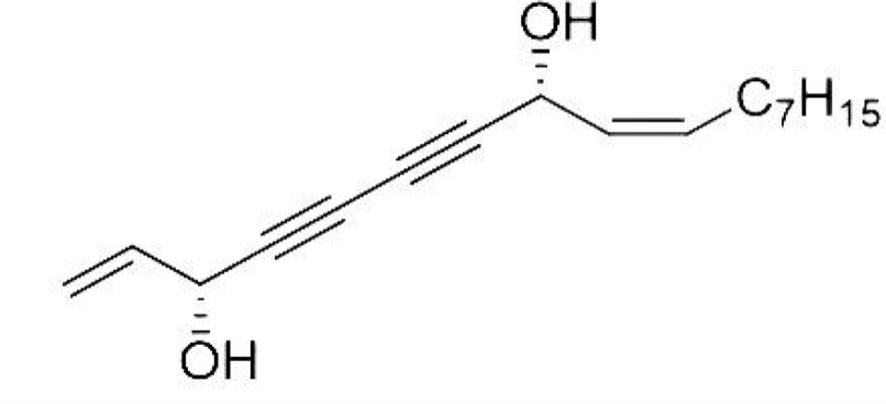
The structure of FADOH.

Natural polyacetylene alcohols can be found in many sources, including natural food products and traditional medicinal materials. Falcarindiol (FADOH, **Figure 1**), a kind of natural polyacetylene alcohol, is a polyacetylenic oxylipin found in vegetables of the carrot family (Apiaceae) and natural Chinese herbal medicines, such as *notopterygium incisum*, *angelica keiskei* and *angelica dahurica*(Yang et al., 2014; Yao and Li, 2015; Andersen et al., 2020; Zhao et al., 2021). It has been found that FADOH has anticancer, antifungal, antibacterial, and anti-inflammatory effects and can also be used as a dietary supplement for neuroprotection (Dawid et al., 2015; Jeon et al., 2020). There are more and more reports on the antifungal activity against plant fungi using polyacetylene alcohols, but there is little research on the antifungal activity against dermatophytes. Previous studies have found that the mechanism of polyacetylene alcohols against plant fungi and tumors is related to lipid metabolism (Nickel et al., 2015; Andersen et al., 2020). We hypothesize that the mechanism of FADOH against dermatophytes is also related to lipid metabolism. This work mainly explores whether the combination of FADOH and ITC exhibits synergistic inhibitory activity *in vitro* against dermatophytes.

## Materials and methods

### Tested strains and culture conditions

Our previous study determined the antifungal activity of FADOH against *Candida* spp., dermatophytes, *Aspergillus* and dematiaceous fungi. We found that FADOH had strong antifungal activity against dermatophytes, so we chose dermatophytes for subsequent experiments. In this study, clinical isolates were tested, including 11 *T. rubrum*, 11 *T. mentagrophytes*, and 6 *M. canis*. As reference strains were used *T. rubrum* ATCC4438 and *T. mentagrophytes* ATCC4439. All strains were typically preserved and subcultured on potato dextrose agar (PDA). Species were all identified by phenotype analysis and DNA sequencing. For molecular identification of common dermatophytes, extracted DNAs were amplified by the universal fungal primers ITS1 (5’-TCCGTA GGTGAACCTGCG G-3’) and ITS4 (5’-TCCTCCGCT TATTGATATGC-3’), and digested with *Mval* enzymes. Final Molecular identification was performed by sequencing and BLAST (http://www.ncbi.nlm.nih.gov/BLAST/) from the National Center for Biotechnology Information (NCBI) database.

### Antifungal agents

All agents (FADOH and ITC) were purchased from Selleck Biotech Co., Ltd. The stock solutions of tested compounds were filter sterilized and prepared following the broth microdilution method M38-Ed3 as detailed by the Clinical and Laboratory Standards Institute (CLSI) (PA, 2017). The stock solutions of FADOH and ITC were dissolved in dimethyl sulfoxide to 20mg/ml and 200μg/ml, respectively. All stock solutions were stored at -20°C.

### Antifungal susceptibility test

The minimum inhibitory concentrations (MICs) of FADOH and ITC in case of tested dermatophyte strains were determined by the broth microdilution method based on the CLSI M38-Ed3 (PA, 2017). The final concentrations of FADOH and ITC from column 2 to column 11 in 96-well plates were 200–0.39μg/ml and 0.5–0.001μg/ml, respectively (100μl). RPMI1640 medium without drug was added to columns 1 and 12 as blank control and growth control, respectively. All strains were subcultured on PDA at 28°C for five days. Conidia were suspended in sterile saline solution (0.85%), and the suspension was twofold more concentrated than the density needed for testing (1×103 to 3×103 CFU/ml). 100µl serially diluted drugs and 100 µl cell suspensions were added to 96-well plates and then incubated at 35°C for four days. The growth inhibition was measured by optical density (OD) at 600 nm (OD600) using a microplate reader (Tecan SUNRISE). The MIC was defined as the concentration of tested compound that inhibited growth and proliferation of dermatophyte cells in 80% comparing to growth control (background OD subtracted). All assays were performed in triplicate and repeated on another day.

### Checkerboard method

For the broth microdilution checkerboard assays, each drug was serially diluted 2-fold in RPMI 1640 medium. The final FADOH and ITC concentrations ranged from 50 to 0.78µg/ml and 0.5 to 0.001µg/ml, respectively. A 50µl aliquot from solutions with the appropriate concentration of ITC and FADOH was added to the wells in the 2nd to 11th columns and the B to H lines, respectively. Row A and column 1 contained the ITC and FADOH alone, respectively. The well at the intersection of row A and column 1 was the drug-free one that performed in 200µl RPMI1640 as blank control. The 12th column served as the growth control. 100µl cell suspensions to a final concentration of 1–3 × 10^3^ CFU/ml were added to each well mentioned above. The plates were incubated at 35°C for four days. Drug interactions were identified based on the fractional inhibitory concentration index (FICI) (Saracino et al., 2022). A synergistic effect is observed when FICI value ≤ 0.5; an additive effect when 0.5 < FICI value ≤ 1; an indifferent effect when 1 < FICI value ≤ 4; and an antagonistic effect when FICI value > 4. The above tests were performed in triplicate and repeated on another day.

### Drop-plate assay

One strain for which the synergistic effect between FADOH and ITC was selected as a representative strain to perform the drop-plate assay. The YPD solid medium plate was divided into sectors, where each sector contained different concentrations of FADOH (12.5–0.78μg/ml) and ITC (0.125–0.001μg/ml) tested. Standardized inoculum of tested dermatophyte strain, the final concentration of 2× 10^3^ CFU/ml, was spotted on individual sectors. The plate was incubated in a 35°C incubator for four days to observe the growth.

### Time-growth curves

To investigate the dynamic inhibitory activity of FADOH and ITC against dermatophytes, OD values of different drug-treated groups were measured, and time-growth curves were graphed. The time-growth curves were determined by a microplate reader in microtiter plates according to the practical steps of A. B. Alio et al. (Alio et al., 2005). Three strains for which the synergistic effect between FADOH and ITC were chosen as the representative strains, including one *T. rubrum*, one *T. mentagrophytes*, and one *M.canis*. Conidia were diluted to the final concentration of 2× 10^3^ CFU/ml with a culture medium (RPMI 1640) and treated with FADOH (0.78μg/ml), ITC (0.016gμg/ml) or exposed to FADOH + ITC (0.78μg/ml + 0.016gμg/ml). The positive control contained 100μl RPMI 1640 medium plus 100μl of the respective isolate. The negative control had 200μl RPMI 1640 medium as the optical density blank. The plates were inoculated at 35°C, and then the OD values were measured at 600 nm after 8h, 16h, 24h, 48h, 72h, 96h, 120h, and 168h of incubating. Each assay was carried out in triplicate.

### Statistical analyses

All experiments were performed three times, and the mean values were recorded as the final result. The SPSS (Statistical Package for the Social Sciences, Chicago, IL) 21 software package was used for the analyses. One-way analysis of variance (ANOVA) was utilized to examine if there were statistically significant differences between the means of different independent groups. *P* < 0.05 indicates that the difference was considered statistically significant.

## Results

### *In vitro* antifungal activities of individual FADOH and ITC used solely against dermatophytes

The MIC values of FADOH against *T. rubrum*, *T. mentagrophytes*, and *M.canis* tested strains were comprised in the range of 1.56–50μg/ml, 1.56–100μg/ml, and 1.56–100μg/ml, respectively. The MIC values of ITC against *T. rubrum*, *T. mentagrophytes*, and *M.canis* were 0.008–0.5μg/ml, 0.002–0.25μg/ml, and 0.002–0.5μg/ml, respectively. All the determined MIC values are summarized in **Table 1**.

**Table 1:** Assessment of the type of interactions between FADOH and ITC based on susceptibility tests performed for tested dermatophyte strains.

Species	No.	MICs(µg/ml)			
		Agent alone		Combination	
		FADOH	ITC	FADOH/ITC	FICI
*T. rubrum*	ATCC4438	6.25	0.0313	0.78/0.004	0.25 (S)
	TR1	25	0.125	3.125/0.0313	0.375 (S)
	TR2	25	0.0625	1.56/0.016	0.313 (S)
	TR3	12.5	0.008	3.125/0.002	0.5 (S)
	TR4	50	0.125	1.56/0.0625	0.53 (AE)
	TR5	25	0.0625	0.78/0.016	0.313 (S)
	TR6	12.5	0.0625	0.78/0.0313	0.53 (AE)
	TR7	25	0.125	0.78/0.0313	0.28 (S)
	TR8	1.56	0.008	1.56/0.002	1.25 (I)
	TR9	12.5	0.0625	3.125/0.016	0.5 (S)
	TR10	50	0.5	25/0.25	1 (AE)
	TR11	25	0.25	0.78/0.0625	0.28 (S)
*T. mentagrophytes*	ATCC4439	100	0.016	12.5/0.008	0.625 (AE)
	TM1	50	0.0313	0.78/0.008	0.266 (S)
	TM2	100	0.0625	1.56/0.0313	0.516 (AE)
	TM3	25	0.25	6.25/0.0625	0.5 (S)
	TM4	50	0.016	1.56/0.008	0.53 (AE)
	TM5	50	0.0313	12.5/0.016	0.75 (AE)
	TM6	25	0.032	1.56/0.008	0.375 (S)
	TM7	3.125	0.008	0.78/0.002	0.5 (S)
	TM8	12.5	0.004	0.78/0.001	0.313 (S)
	TM9	6.25	0.008	1.56/0.001	0.375 (S)
	TM10	3.125	0.016	0.78/0.004	0.5 (S)
	TM11	1.56	0.002	0.78/0.001	1 (AE)
*M. canis*	MC1	3.125	0.016	0.78/0.004	0.5 (S)
	MC2	1.56	0.004	0.78/0.002	1 (AE)
	MC3	1.56	0.002	0.78/0.001	1 (AE)
	MC4	1.56	0.002	1.56/0.001	1.5 (I)
	MC5	25	0.5	25/0.5	2 (I)
	MC6	100	0.25	50/0.25	1.5 (I)

The MIC value was defined as the concentration of tested compound that inhibited growth and proliferation of dermatophyte cells in 80% comparing to growth control. S, synergistic effect (FICI≤ 0.5); AE, additive effect (0.5 < FICI value ≤ 1); I, indifferent effect (1 < FICI value ≤ 4).

### Checkerboard assay to determine type of interactions between tested antifungals

The synergistic effect between FADOH and ITC is shown in **Table 1** based on the checkerboard assay to assess the susceptibility of different dermatophyte strains. Combining FADOH and ITC reduced the MIC values for these two drugs to 0.78–50μg/ml and 0.001–0.5μg/ml, respectively, exhibiting synergistic and additive activity against 53.3% and 33.3% of all tested dermatophytes, respectively. Among 12 *T. rubrum* strains, synergistic effect, additive effect, and indifferent effect could be observed between tested compounds in eight strains (66.7%), three strains (25%), and one strain (8.3%), respectively. In 12 *T. mentagrophytes* strains, synergistic effect and additive effect could be observed between tested compounds in seven strains (58.3%) and five strains (41.7%), respectively. As for 6 *M. canis* strains, synergistic effect, additive effect, and indifferent effect could be observed between tested compounds in one strain (16.7%), two strains (33.3%), and three strains (50%), respectively.

**Figure 2:**
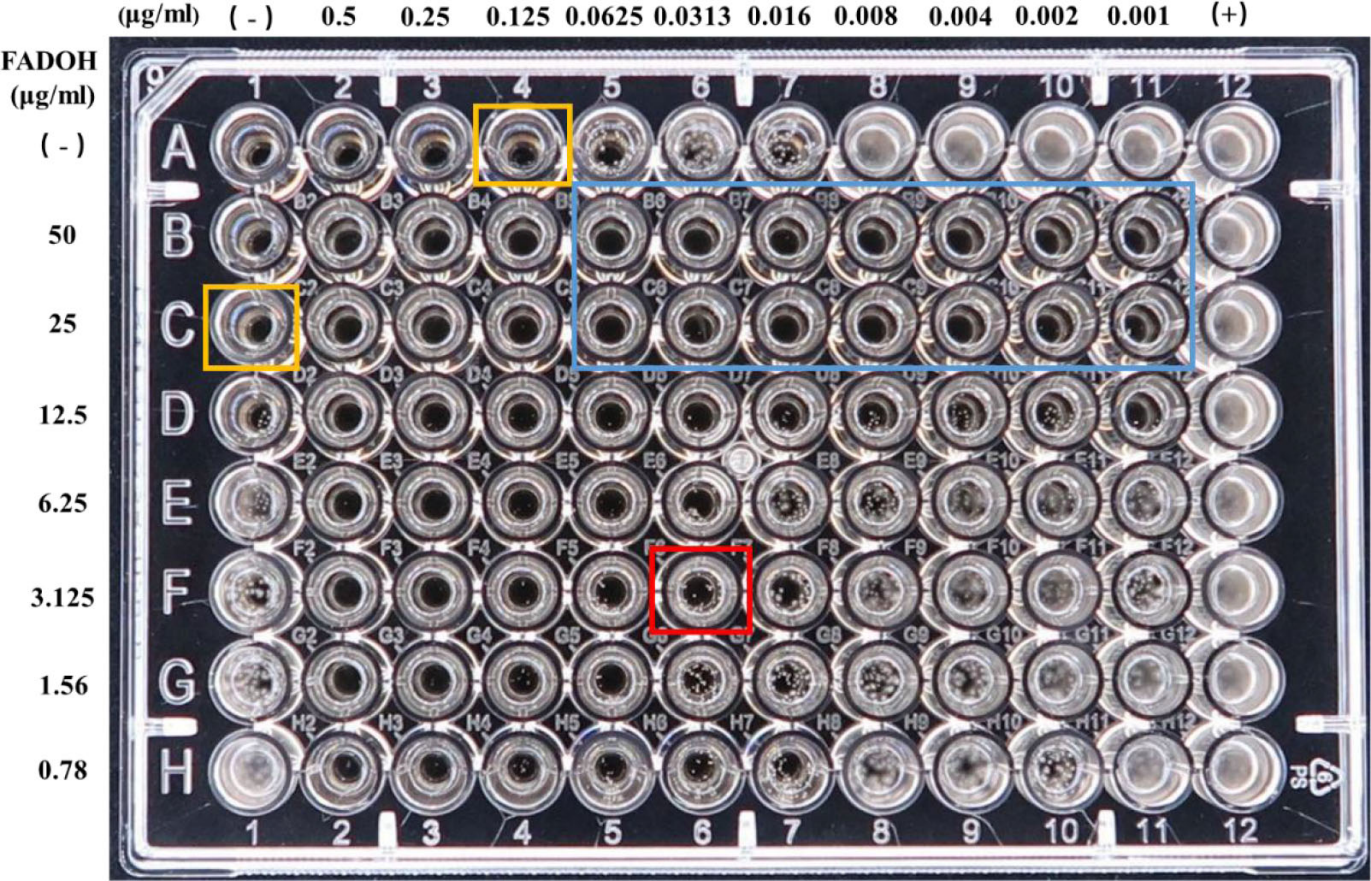
The MIC values determined for FADOH and ITC used solely and in different combinations in the checkerboard assay for *T. rubrum* TR1.

Taking the *T. rubrum* TR1 as a representative strain, the MIC results of the checkerboard assay are shown in **Figure 2**. This figure showed that the MIC values for FADOH and ITC used solely were equal to 25μg/ml and 0.125μg/ml (yellow box), respectively. The combination of FADOH and ITC decreased the MIC values to 3.125μg/ml and 0.0313μg/ml (red box), respectively, showing synergistic activity (FICI=0.375). It could be observed that the combination of FADOH at higher concentrations with ITC, used even at the lowest tested concentrations, strongly inhibited cell growth and proliferation (blue box).

### Drop-plate assay to visualize synergistic effect between FADOH and ITC

**Figure 3:**
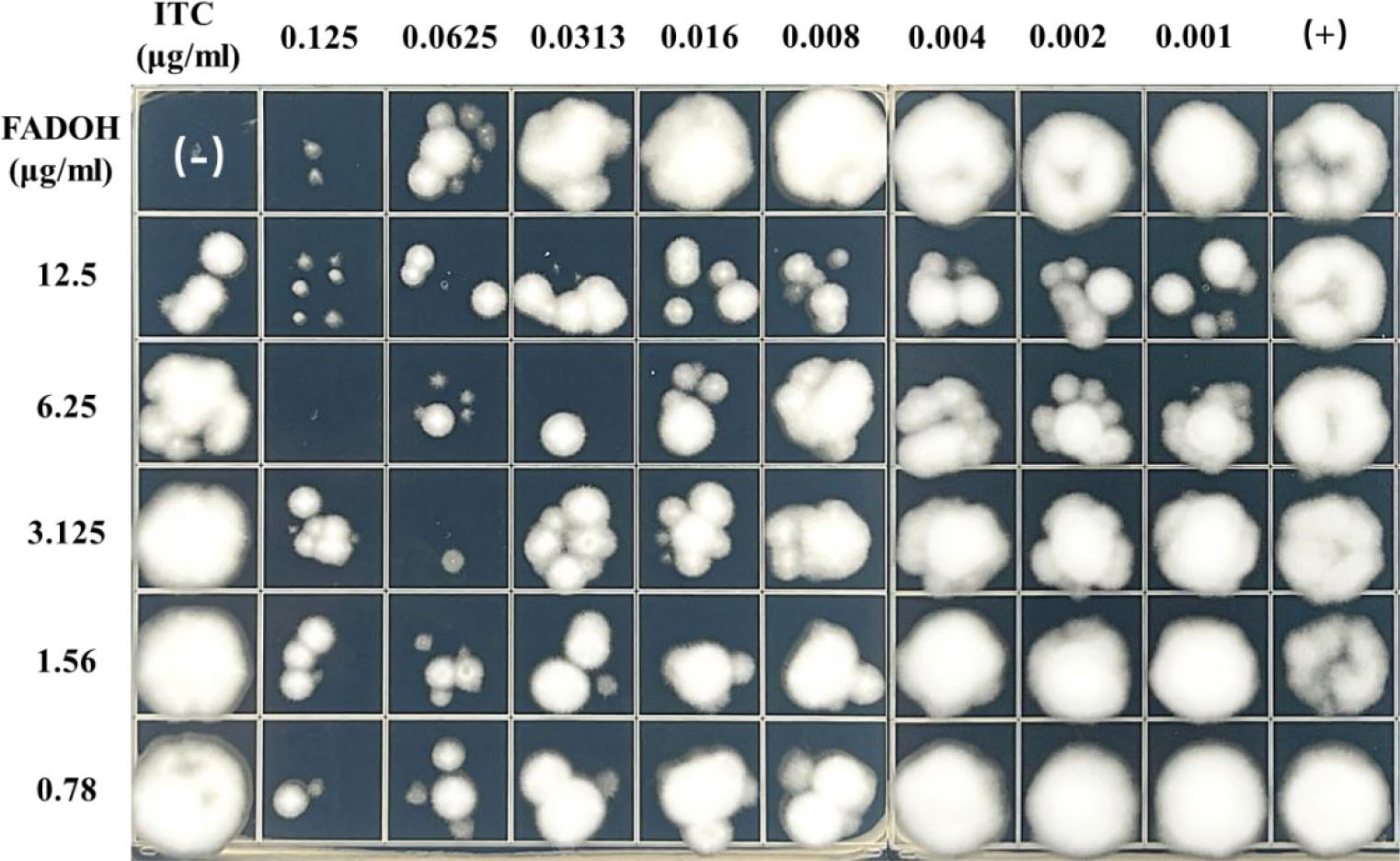
The antifungal activity of FADOH and ITC used solely and in combination toward *T. mentagrophytes* TM10 was assessed based on the dope-plate assay.

To show the synergistic antifungal activity of FADOH and ITC more clearly, a representative strain, *T. mentagrophytes* TM10, was selected for the drop-plate assay. The result showed after combining with FADOH, the decreased growth of fungi indicated that the antifungal activity of ITC was significantly enhanced (**Figure 3**). Especially when combined with 12.5 μg/ml and 6.25μg/ml of FADOH, even the low concentration of ITC still had a strong inhibitory effect on the strain.

### Time-growth curves

**Figure 4:**
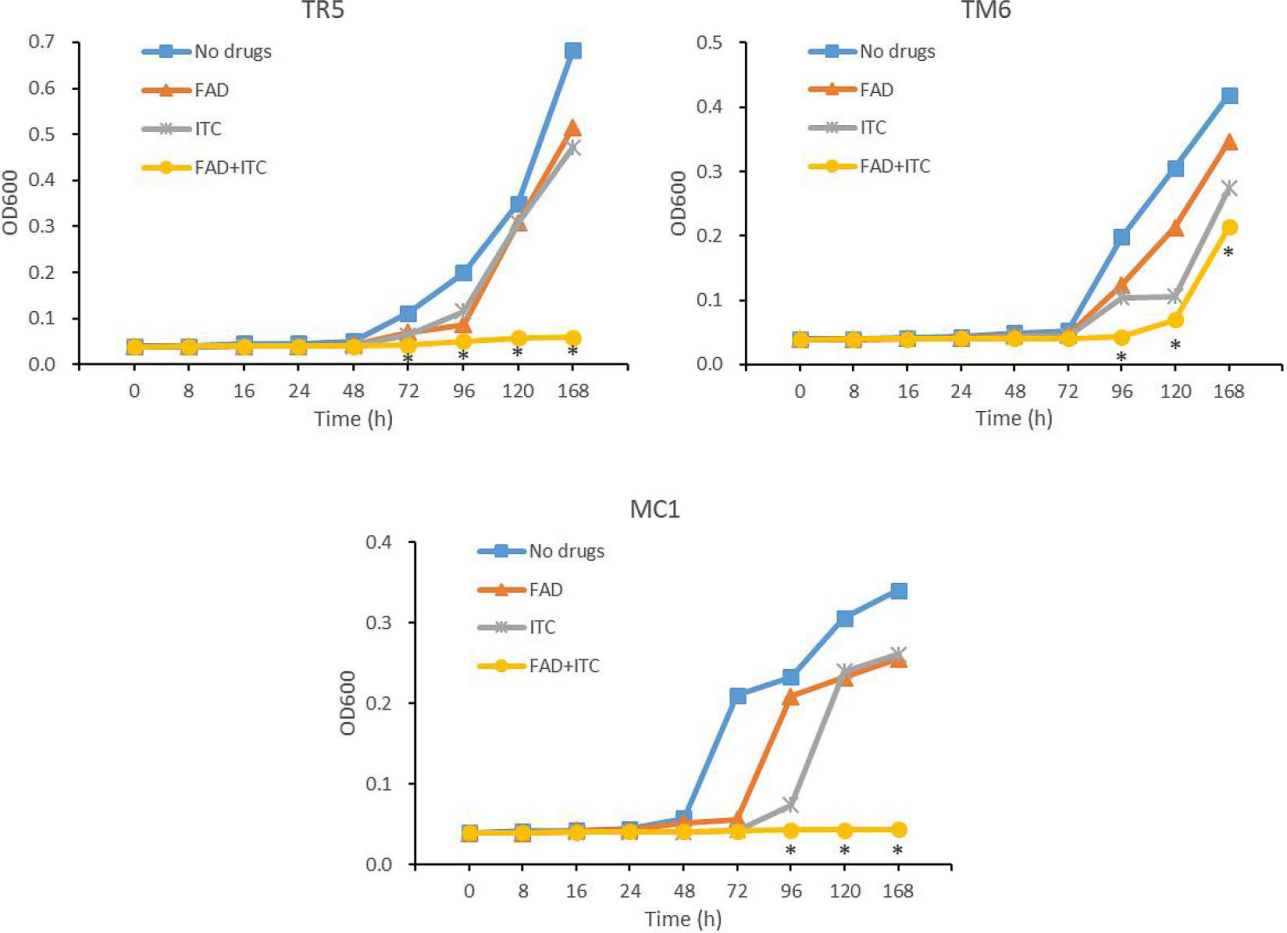
Growth curves of *T. rubrum* TR5, *T. mentagrophytes* TM6, and *M.canis* MC1 after the treatment of FADOH, ITC, and the combination of FADOH and ITC, which was measured by optical density (OD at 600 nm). *P < 0.05, the combination group showed a statistically significant difference from the control group, FADOH group, and ITC group.

Time-growth curves were fitted to further understand the dynamic suppressive action of FADOH on ITC against dermatophytes. We took *T. rubrum* TR5, *T. mentagrophytes* TM6, and *M.canis* MC1 as the representative strains. The results showed that the combination of FADOH and ITC against all three strains demonstrated a potent synergistic antifungal effect at 96h, which was still very pronounced from 96h to 168h (**Figure 4**).

## Discussion

Immunosuppressed patients have a higher incidence of superficial dermatophytosis and are more prone to develop devastating invasive dermatophyte infection (Marconi et al., 2010). However, antifungal drugs for treating severe invasive fungal infections are still limited (Revie et al., 2018). Moreover, the frequent and preventive use of antifungal drugs has led to the development of antifungal drug resistance (Revie et al., 2018). The four main classes of antifungal drugs are polyenes, azoles, allylamines and echinocandins. Azole antifungal agents, such as fluconazole and ITC, have been widely used in treating dermatophytosis. Unlike allylamines (e.g., terbinafine), azoles could potentiate drug resistance development in dermatophytes (Ghannoum, 2016). Combination therapy might effectively treat severe refractory fungal infections (Xu et al., 2019). It has been found that the combination of new antifungal drugs and traditional antifungal drugs has certain synergistic or at least additive activity against many fungi *in vitro* (Vazquez, 2007). Natural Chinese herbal medicine contains complex constituents with good safety and a broad antifungal spectrum, including *Candida, Cryptococcus neoformans* and all kinds of plant fungi (Tian et al., 2010; Zhang et al., 2020; Yang et al., 2022). In addition to the main components, there are a lot of unknown trace components with novel structures and strong biological activity, which are considered as one of the important therapeutic substances and a huge source of innovative drugs of traditional Chinese medicine (Xia et al., 2022). Chinese herbal medicine can be used as a resource for the detection of new antifungal targets and has become an essential research focus and focal point in recent years.

The antifungal activity of polyacetylene alcohols isolated from apiaceous vegetables and Chinese herbal medicine has recently attracted attention. Yoon et al. found that polyacetylalcohol ciryneol A had good antifungal activity against plant fungi, such as *Magnaporthe oryzae*, *Colletotrichum coccodes*, and *Pythium ultimum*, with IC50 values below 50μg/ml (Kim et al., 2011). Lecomte M et al.found that FADOH had a good inhibitory effect on the fungal leaf blight pathogen *Alternaria alternata* (IC50 ranging from 34 to 102 μM) (Lecomte et al., 2012). Liu et al. found that C18 polyacetylenes from *panax stipuleanatus* exhibited high potencies against eight pathogenic fungal species tested, such as *Colletotrichum gloeosporioiles*, *Fusarium graminearum*, and *Fusarium pseudograminearum*, with half-maximum effective concentrations ranging from 8 to 425μg/ml (Liu et al., 2020). However, no antifungal effects of polyacetylene alcohols against dermatophytes have been reported so far.

Our study showed that FADOH had a specific antifungal effect against dermatophytes, with MICs ranging from 1.56 to 100μg/ml. FADOH combined with ITC had good synergistic and additive activity against most dermatophytes (86.7%), particularly *T.rubrum* and *T. mentagrophytes*. Interestingly, FADOH and ITC had synergistic or additive effects against all *T. mentagrophytes*. In addition, FADOH could reduce the MIC values of ITC against most dermatophytes (93.3%) to 2-8 times. Furthermore, drop-plate assay verified the synergistic activity of FADOH and ITC visually. We selected one strain per species for the time-growth curve test to verify the dynamic synergistic effect of FADOH and ITC on dermatophytes. The time-growth curves showed that the combination of FADOH and ITC against all three strains demonstrated a potent synergistic antifungal effect at 96h, which was still very pronounced from 96h to 168h. The combination of the two drugs against *T. rubrum* TM5 showed an obvious synergistic antifungal effect at 72 hours. Unlike *T. rubrum* TR5 and *M. canis* MC1, *T. mentagrophytes* TM6 started to grow similarly like in the control after 120h. As for whether the combination of FADOH and ITC against *T. rubrum* shortens the onset time of antifungal activity and whether the synergistic inhibitory effect on *T. mentagrophytes* is not as strong as the other two dermatophytes, we need to conduct time-growth curve tests on more strains in the future for further verification.

Although there is no report on the anti-dermatophyte mechanism of natural polyacetylene alcohols, some reports have been made on its anti-plant fungus and antitumor mechanism. HR Jin et al. found that FADOH exerted its antitumor activity by inducing endoplasmic reticulum (ER) stress and apoptosis (Jin et al., 2012). Proteomics also found that polyacetylene and other compounds were mainly involved in the metabolism of lipids and fatty acids, interfering with the activities of lipid-modifying enzymes and various membrane-related proteins such as endoplasmic reticulum (Nickel et al., 2015). CB Andersen et al. found that FADOH could activate the expression of sterol transporter, causing the redistribution of lipids and increased formation of lipid droplets in cells, which led to endoplasmic reticulum stress and cell death (Andersen et al., 2020). Fungi and human cells are eukaryotes, and sterols are the membrane lipids of most eukaryotes (Chen et al., 2007). The change of sterol in the cell membrane will destroy the integrity and fluidity of the cell membrane and affect the expression of membrane protein, thus affecting the growth and metabolism of cells (Jorda and Puig, 2020). Sterols in fungi are called ergosterol, a vital component of the fungal plasma membrane (Shapourzadeh et al., 2016). Significant structural differences in fatty acid between fungi and mammalian cells exist, making it a promising antifungal target (Bitencourt et al., 2013). The fungal cytochrome P450 enzyme sterol 14α-demethylase is a key enzyme in the ergosterol biosynthesis pathway and is the target of azoles (Rosam et al., 2020). Therefore, we speculate that the synergistic mechanism of polyacetylene alcohols and azoles against dermatophytes may mainly affect the metabolism of ergosterol and lipid, induce endoplasmic reticulum stress, and thus lead to fungal cell apoptosis.

Although further work is needed to explore the underlying mechanism for the observed synergistic activity, our findings demonstrate the potential activity of FADOH as an antifungal adjuvant to enhance the antifungal activity of azoles against dermatophytes.

## Conclusions

In summary, FADOH combined with ITC has synergistic and additive antifungal activity against most dermatophytes, indicating that natural polyacetylene alcohols may be a more reliable and safe combination therapy for treating dermatophytoses and invasive dermatophyte infection.

## Data availability statement

The original contributions presented in the study are included in the article/supplementary material. Further inquiries can be directed to the corresponding author.

## Author contributions

SK carried out the *in vitro* antifungal experiment and wrote the manuscript. ZG, SL, and HY collected and analyzed the experimental data. LA, JT, QC, and RN designed and interpreted the experiment data. LY revised the manuscript critically for important content. All authors contributed to the article and approved the submitted version.
